# Knowledge, attitudes, practices, and perception of COVID-19 preventive measures among adult residents of Matadi (Democratic Republic of the Congo) after the third epidemic wave

**DOI:** 10.3389/fpubh.2024.1363717

**Published:** 2024-06-28

**Authors:** Yannick Munyeku-Bazitama, Patient Okitale-Talunda, Justus Nsio-Mbeta, Patrick Mpingabo-Ilunga, Paul Tshiminyi-Munkamba, Aimé Umba-Phuati, Jacques Kimfuta, Ferdinand Ango-Phukuta, Goethe Makindu, Raymond Mufwaya-Nsene, Ryoko Asari, Saeda Makimoto, Steve Ahuka-Mundeke, Mitsuo Isono, Sheila Makiala-Mandanda, Jean-Jacques Muyembe-Tamfum

**Affiliations:** ^1^Département de Virologie, Institut National de Recherche Biomédicale, Kinshasa, Democratic Republic of the Congo; ^2^Division of Global Epidemiology, International Institute for Zoonosis Control, Hokkaido University, Sapporo, Japan; ^3^Département de Biologie Médicale, Faculté de Médecine, Université de Kinshasa, Kinshasa, Democratic Republic of the Congo; ^4^Direction de Surveillance Epidémiologique, Ministère de la Santé, Hygiène et Prévention, Kinshasa, Democratic Republic of the Congo; ^5^Division Provinciale de Santé du Kongo Central, Matadi, Democratic Republic of the Congo; ^6^Japan International Cooperation Agency, Tokyo, Japan; ^7^Departments of Virology and Parasitology, Graduate School of Medicine, Osaka Metropolitan University, Osaka, Japan

**Keywords:** COVID-19, knowledge, attitudes, practices, perception, prevention, vaccine, noncompliance

## Abstract

**Background:**

Several governments from African countries, including the Democratic Republic of the Congo (DRC), implemented stringent public health measures to curb COVID-19 transmission in the early phases of the pandemic. While these restrictive measures are believed to have contributed to lowering case incidence and related mortality in DRC, data on the population’s knowledge and adherence are limited. This study aimed to assess the knowledge, perception, attitudes, and practices of COVID-19 preventive measures and associated factors among adult residents of Matadi, thereby generating evidence for a strategy adjustment as the COVID-19 response is transitioning from emergency to control status.

**Methods:**

We used data from a population-based cross-sectional study conducted in October 2021. Consenting participants were enrolled through a multi-stage cluster sampling approach and administered a pre-tested structured questionnaire using a mobile application (Epicollect 5). We analyzed adult participants’ data using STATA 15.1. Univariable and multivariable analyses were applied to identify factors associated with good knowledge, good perception, positive attitude and good practice.

**Results:**

We included 1,269 adult respondents for the secondary analysis. One respondent in six was female. The median age was 36 years (IQR 24–50). Most respondents (76.5%) had good knowledge. Respondents aged 40–49 years and those with vocational education level were 1.7 time (AOR 1.75, 95% CI 1.07–2.87) and twice as likely (AOR 2.06, 95% CI 1.01–4.21) to have good knowledge. Preventive measures were perceived as efficient by 45% of respondents. Good perception was associated with education level, profession, average household monthly income and good knowledge. Only 40% of respondents had a positive attitude. A positive attitude was associated with age, education level, and good knowledge. Respondents having good practice represented 5.8%. Good practice was associated with good knowledge, attitude and perception.

**Conclusion:**

Most respondents were knowledgeable, had a good perception of government-related COVID-19 preventive measures, a moderately positive attitude and an extremely low level of good practice. Current COVID-19 preventive strategies, including vaccination rollout, need adjustment into high-efficiency, context-based and risk group-specific interventions. Evidence generated by this study will improve preparedness and response to future outbreaks.

## Introduction

The coronavirus disease (COVID-19) is an emerging disease caused by the severe acute respiratory syndrome coronavirus-2 (SARS-CoV-2) (1). As of November 9, 2023, 771,679,618 cases were reported worldwide, including 6,977,023 deaths (2). COVID-19 was declared a global pandemic by the World Health Organization (WHO) in March 2020 (2). Thereafter, several models predicted COVID-19 incidence and mortality would be higher in Africa given the numerous challenges faced by the healthcare systems of most African countries (3). In addition, COVID-19 vaccines, considered from the earliest phases of the pandemic as essential tools for pandemic control, were hardly available to African countries due to poor research and development capacities and vaccine nationalism (4, 5). Governments from several African countries opted for more feasible and less costly preventive measures based on previous experiences dealing with infectious disease outbreaks such as Ebola Virus Disease (6). Therefore, most governments implemented stringent public health measures to contain COVID-19 and curb transmission during the early phases of the pandemic. These measures included partial or complete lockdowns with the closure of international airports, schools and congregation points, curfew, prohibition of mass gatherings, downsizing public and private transport, and non-pharmaceutical interventions (3). The latter included mainly physical distancing, facemask wearing, avoiding crowded areas, frequent handwashing with soap, ventilation of indoor spaces, and teleworking to some extent (7). Altogether, these measures have contributed to lowering COVID-19 case incidence during the early phases of the pandemic in Africa (3). Later, these measures were progressively lifted because of their negative impact on the economy and trade. As a result, SARS-CoV-2 extensively spread, especially with the emergence of highly contagious SARS-CoV-2 variants (8, 9). Particularly, in the Democratic Republic of the Congo (DRC), the lightning of stringent public health measures after the first wave and the spread of the SARS-CoV-2 Beta variant (B.1.351) during the second wave led to a 4-fold increase in seroprevalence between the first and the second epidemic waves (16.6% vs. 76.5%) (10). While implementing restrictive COVID-19 preventive measures seems to have contributed to lowering the incidence of severe COVID-19 and related mortality in DRC (11), data on the population’s adherence to these government measures is scanty (12, 13). Adherence to preventive measures is affected by the population’s knowledge, attitudes, practice and perception (KAPP) (14, 15). Thus, understanding the population’s KAPP helps to identify gaps in the implementation of public health interventions, factors associated with poor adherence, and groups requiring more specific approaches. As the COVID-19 epidemic evolves, there is a critical need to adjust public health interventions into tailored preventive and control measures so as to prepare for future outbreaks.

In DRC, most COVID-19 studies have been conducted in Kinshasa (10, 12, 16, 17), the capital and epidemic epicenter, leaving behind cities such as Matadi in the neighboring Kongo Central province, which has sustained economic exchanges with Kinshasa. Despite the geographical proximity and trade between these two cities, the KAPPs of their respective populations regarding government preventive measures may differ. Matadi residents, including some rural communities, may consider themselves less at risk of infection and adhere less to government preventive measures. Therefore, developing mitigation strategies in Matadi based on evidence generated from Kinshasa could hamper efforts to control the epidemic in the context of limited available resources. This study aimed to assess the knowledge, attitudes, practices, and perception of government-related COVID-19 preventive measures among adult residents of Matadi and to provide insights into associated factors that should be considered to adjust the local response strategies and develop future ones.

## Methods

### Study design and setting

We used data from a population-based cross-sectional study conducted in Matadi from 16 October to 24 October 2021, after the third wave of the COVID-19 epidemic (11). The primary study aimed at measuring SARS-CoV-2 infection seroprevalence and associated factors. Matadi is the main seaport city of DRC and the political capital of the Kongo Central Province. It is located 352 km from Kinshasa and has an estimated population of 402,397 living in two health districts: Matadi (55%) and Nzanza (45%) (18, 19).

### Study population and sample size

The minimum required sample size for the primary study was calculated considering an expected seroprevalence of at least 15%, a precision of 1%, a design effect of 2, and a nonresponse rate of 30% (11). A total of 2,241 participants were enrolled, including 1,602 adult participants (≥18 years of age), using a multi-stage cluster sampling as described elsewhere (11). Only 1,269 (79.2%) of all adult participants agreed to answer questions on government-related COVID-19 preventive measures and were considered for secondary data analysis. The minimum required sample size for the secondary analysis was 384 based on an estimated 50% proportion of adult residents having good knowledge, good perception, positive attitude and good practice toward COVID-19 preventive measures.

### Data collection

A structured pre-tested questionnaire was administered to participants using a mobile application (Epicollect 5, Imperial College, London) as part of the primary study. The questionnaire was adapted following recommendations from WHO, the Ministry of Health, and a survey conducted in Kinshasa (12, 20). It included socio-demographic characteristics, current and past medical history, COVID-19 vaccination history, exposure to SARS-CoV-2, and COVID-19-related behavioral characteristics. Additionally, we collected data on participants’ knowledge, attitudes, practices, and perception of government-related COVID-19 control measures on which this secondary data analysis focused. The questionnaire was translated into local languages and administered by trained and experienced surveyors.

### Key variables and assessment

#### Outcome variables

##### Knowledge of government-related COVID-19 preventive measures

To assess knowledge of government-related COVID-19 preventive measures, study participants were asked whether they knew the following seven measures: curfew, mandatory mask-wearing, quarantine, lockdown, public spaces closure, mass gathering prohibition and limitation of public transportation use. For each assessed measure, one point was assigned when a participant knew the assessed measure, while 0 point was assigned when the measure was unknown or the participant was unsure.

##### Perception of government-related preventive measures efficiency

Participants’ perception of government-related COVID-19 preventive measures’ efficiency was assessed using four response options, namely “not efficient”, “not sure or undecided”, “somewhat efficient”, “efficient”.

##### Attitudes toward COVID-19

Participants’ attitude toward COVID-19 was measured by their willingness to get the vaccine and get tested in case they experience flu-like symptoms. We used a zero to four-point Likert scale to assess each component of participants’ attitudes with the following scoring: 0 (not willing), 1 (somewhat not willing), 2 (undecided), 3 (somewhat willing), and 4 (willing).

##### Practice of government-related COVID-19 preventive measures

To measure the practice of government-related COVID-19 preventive measures, study participants were asked whether they practiced the following nine measures: wearing a facemask, wearing gloves, washing hands with soap, washing hands for at least 20 s, staying at home, avoiding crowded spaces, keeping 1.5 m distance, washing clothes upon returning home, and reducing public transportation use. For each measure, one point was assigned when a participant practiced the measure, while 0 was assigned when he did not.

##### Explanatory variables

Seven explanatory variables were considered in this secondary data analysis: sex, health district, age, education, household average monthly income, occupation, and religion.

### Statistical analyses

We used STATA 15.1 (Stata Corp LLC, College Station, TX, United States) for data analysis. Proportions were used to summarize categorical variables while the median with the interquartile range was used to summarize continuous variables. The knowledge, attitude, and practice were summarized by transforming the point scale to percentages and categorized using Bloom’s cut-off-points as good/positive (80–100%), moderate/neutral (60–70%), and poor/negative (less than 60%) (14). Associations between outcomes and explanatory variables were assessed using logistic regression.

For logistic regression analyses, knowledge, attitude and practice were further categorized into two groups by considering percentages above 60 as good/positive and percentages below 60 as poor/negative. Similarly, the perception was classified as good if a respondent thought that COVID-19 preventive measures efficiently limited the disease spread.

Differences between groups were assessed using the Fisher’s Exact test or the paired t-test. We considered a *p*-value of <0.05 statistically significant.

## Results

### Sociodemographic characteristics

Overall, 1,269 adult participants from 444 households were included in the secondary data analysis, of which 57.8% (733/1,269) were recruited from the Matadi health district and 42.2% (536/1,269) from the Nzanza health district. One participant out of six was female (61.0%). The median age was 36 (interquartile range 24–50). Participants aged 18 to 28 represented one-third of the respondents (34.4%). More than half participants (54.2%) had achieved secondary education, whereas 5.9% had no formal education and nearly one-fifth (19.6%) had university-level education. Most participants (91.7%) lived in households with a monthly average income between US $ 1 and 250. Nearly nine participants in 10 (87.5%) were Christian (Table 1).

**Table 1:** Sociodemographic characteristics of study participants.

Variables (*N* = 1,269)	*n*	%	Median	IQR*
Sex				
Female	774	61.0		
Male	495	39.0		
Health district				
Matadi	733	57.8		
Nzanza	536	42.2		
Age group in years			36	24–50
18–28	437	34.4		
29–39	274	21.6		
40–49	248	19.5		
> 50	310	24.4		
Education				
No formal education	75	5.9		
Primary	111	8.8		
Secondary	688	54.2		
Vocational	146	11.5		
University	249	19.6		
Household average monthly income (US $)				
1–50	495	39.0		
51–250	669	52.7		
251–500	79	6.2		
501–1,000	26	2.1		
Occupation				
Unemployed	134	10.6		
Housewife	240	18.9		
Student	231	18.2		
Public servant	40	3.2		
Private sector	403	31.8		
Healthcare worker	221	17.4		
Religion				
Christian	1,110	87.5		
Traditional	115	9.1		
Other	44	3.5		
*IQR: Interquartile range				

### Knowledge of government-related COVID-19 preventive measures

Three respondents in four (75.6%) had good knowledge. In contrast, nearly one-fifth (19.8%) had poor knowledge and 4.6% had average knowledge. Most respondents heard about the COVID-19 vaccine (76.8%) and only 36.3% knew the location of the nearest COVID-19 testing center. Regarding specific knowledge questions, at least 80% of respondents knew COVID-19 preventive measures recommended by the government, though nearly four respondents in 10 (38.9%) did not recognize lockdown as one of these measures (Table 2).

**Table 2:** Participants’ knowledge of government-related COVID-19 preventive measures.

Knowledge questions (*N* = 1,269) Have any of the following measures been recommended by the government to stop the spread of COVID-19?	*n*	%
Curfew		
No	53	4.2
Yes	1,216	95.8
Facemask wearing		
No	69	5.4
Yes	1,200	94.6
Quarantine		
No	218	17.2
Yes	1,051	82.8
Lockdown		
No	493	38.9
Yes	776	61.1
Closure of public areas		
No	205	16.1
Yes	1,064	83.9
Prohibition of mass gatherings		
No	251	19.8
Yes	1,018	80.2
Limitation of public transportation use		
No	252	19.9
Yes	1,017	80.1

The multivariable analysis of data revealed that being 40–49 years of age and having a vocational education were associated with good knowledge. Respondents 40–49 years of age had 75% increased odds of having a good knowledge than those 18–28 years of age (AOR 1.75, 95% CI 1.07–2.87, *p* = 0.025). Similarly, the odds of having good knowledge were twice as high among respondents with a vocational education than those with no formal education (AOR 2.06, 95% CI 1.01–4.21, *p* = 0.047; Table 3).

**Table 3:** Factors associated with participants’ knowledge of government-related COVID-19 preventive measures.

Variables (*N* = 1,269)	*N*	Knowledge		OR	*p*	AOR	*p*
		Good, *n* (%)	Poor, *n* (%)				
Sex							
Female	774	602 (77.8)	172 (22.2)	1		1	
Male	495	416 (84.0)	79 (16.0)	1.50 (1.12–2.02)	0.007	1.29 (0.94–1.80)	0.114
Health district							
Matadi	733	574 (78.3)	159 (21.7)	1		1	
Nzanza	536	444 (82.8)	92 (17.2)	1.34 (1.01–1.78)	0.046	1.23 (0.90–1.67)	0.191
Age group in years							
18–28	437	350 (80.1)	87 (19.9)	1		1	
29–39	274	205 (74.8)	69 (25.2)	0.74 (0.52–1.06)	0.099	0.89 (0.58–1.37)	0.599
40–49	248	213 (85.9)	35 (14.1)	1.51 (0.99–2.32)	0.058	1.75 (1.07–2.87)	0.025
> 50	310	250 (80.6)	60 (19.4)	1.04 (0.72–1.49)	0.851	1.14 (0.73–1.78)	0.562
Education							
No formal education	75	52 (69.3)	23 (30.7)	1		1	
Primary	111	88 (79.3)	23 (20.7)	1.69 (0.86–3.31)	0.125	1.66 (0.82–3.35)	0.156
Secondary	688	545 (79.2)	143 (20.8)	1.69 (0.99–2.84)	0.051	1.50 (0.85–2.64)	0.158
Vocational	146	123 (84.3)	23 (15.7)	2.36 (1.21–4.58)	0.011	2.06 (1.01–4.21)	0.047
University	249	210 (84.3)	39 (15.7)	2.38 (1.31–4.33)	0.004	1.80 (0.93–3.47)	0.079
Household average monthly income (US $)							
1–50	495	399 (80.6)	96 (19.4)	1		1	
51–250	669	528 (78.9)	141 (21.1)	0.90 (0.67–1.20)	0.481	0.83 (0.62–1.12)	0.230
251–500	79	67 (84.8)	12 (15.2)	1.34 (0.69–2.58)	0.376	1.17 (0.59–2.31)	0.642
501–1,000	26	24 (92.3)	2 (7.7)	2.89 (0.67–12.4)	0.155	2.45 (0.55–10.9)	0.240
Occupation							
Unemployed	134	102 (76.1)	32 (23.9)	1		1	
Housewife	240	186 (77.5)	54 (22.5)	1.08 (0.66–1.78)	0.761	0.99 (0.57–1.69)	0.958
Student	231	192 (83.1)	39 (16.9)	1.54 (0.91–2.61)	0.105	1.47 (0.81–2.67)	0.207
Public servant	40	36 (90.0)	4 (10.0)	2.82 (0.93–8.54)	0.066	2.12 (0.68–6.67)	0.197
Private sector	403	312 (77.4)	91 (22.6)	1.08 (0.68–1.71)	0.756	0.96 (0.59–1.58)	0.883
Healthcare worker	221	190 (85.7)	31 (14.0)	1.92 (1.11–3.33)	0.020	1.41 (0.77–2.57)	0.262
Religion							
Christian	1,110	904 (81.4)	206 (18.6)	1		1	
Traditional	115	84 (73.0)	31 (27.0)	0.62 (0.39–0.96)	0.098	0.68 (0.43–1.07)	0.098
Other	44	30 (68.2)	14 (31.8)	0.49 (0.25–0.94)	0.057	0.51 (0.26–1.02)	0.057

### Perception of government-related COVID-19 preventive measures efficiency

Overall, 45.0% of respondents perceived the government’s COVID-19 preventive measures as efficient in limiting the spread of COVID-19 in DRC. Nearly 39% of respondents perceived these measures as somewhat efficient, 10.9% were unsure, and 5.2% thought they were inefficient. Respondents from the Nzanza health district were 43% less likely to perceive COVID-19 preventive measures as efficient than respondents from the Matadi health district (AOR 0.57, 95% CI 0.44–0.74, *p* = 0.000; Table 4). Similarly, respondents with primary, secondary, vocational, and university education were, respectively, 53% (AOR 0.47, 95% CI 0.25–0.90, *p* = 0.023), 56% (AOR 0.44, 95% CI 0.26–0.77, *p* = 0.004), 78% (AOR 0.22, 95% CI 0.11–0.42, *p* = 0.000) and 57% (AOR 0.43, 95% CI 0.24–0.79, *p* = 0.006) less likely to perceive COVID-19 preventive measures as efficient than respondents with no formal education. However, we observed a trend toward increased odds of perceiving preventive measures as efficient with household average monthly income. Respondents from households with an average monthly income of US $ 51–250, US $ 251–500, and US $ 501–1,000 were, respectively, 1.7 (AOR 1.74, 95% CI 1.35–2.24, *p* = 0.000), 3.8 (AOR 3.89, 95% CI 2.29–6.64, *p* = 0.000), and 54.2 times (AOR 54.2, 95% CI 7.12–413, *p* = 0.000) more likely to perceive COVID-19 preventive measures as efficient than respondents from households with an average monthly income of US $ 1–50 (Table 4). Similarly, respondents with good knowledge were twice as likely to perceive preventive measures as efficient (AOR 2.49, 95% CI 1.82–3.43, *p* = 0.000). Regarding respondents’ occupation, students (AOR 1.79, 95% CI 1.07–3.01, *p* = 0.026) and public servants (AOR 2.28, 95% CI 1.06–4.93, *p* = 0.035) were more likely to perceive COVID-19 preventive measures as efficient than unemployed respondents.

**Table 4:** Factors associated with participants’ perception of government-related COVID-19 preventive measures efficiency.

Variables (*N* = 1,269)	*N*	Perception		OR	*p*	AOR	*p*
		Good, *n* (%)	Poor, *n* (%)				
Sex							
Female	774	336 (43.4)	438 (56.6)	1		1	
Male	495	235 (47.5)	260 (52.5)	1.18 (0.94–1.48)	0.156	1.01 (0.77–1.31)	0.951
Health district							
Matadi	733	358 (48.8)	375 (51.2)	1		1	
Nzanza	536	213 (39.7)	323 (60.3)	0.69 (0.55–0.87)	0.001	0.57 (0.44–0.74)	0.000
Age group in years							
18–28	437	189 (43.3)	248 (56.7)	1		1	
29–39	274	121 (44.2)	153 (55.8)	1.04 (0.76–1.41)	0.812	1.08 (0.73–1.60)	0.681
40–49	248	113 (45.6)	135 (54.4)	1.09 (0.80–1.50)	0.558	1.19 (0.80–1.79)	0.381
> 50	310	148 (47.7)	162 (52.3)	1.20 (0.89–1.60)	0.224	1.31 (0.89–1.94)	0.162
Education							
No formal education	75	45 (60.0)	30 (40.0)	1		1	
Primary	111	52 (46.8)	59 (53.2)	0.59 (0.32–1.06)	0.079	0.47 (0.25–0.90)	0.023
Secondary	688	306 (44.5)	382 (55.5)	0.53 (0.33–0.87)	0.011	0.44 (0.26–0.77)	0.004
Vocational	146	47 (32.2)	99 (67.8)	0.32 (0.18–0.56)	0.000	0.22 (0.11–0.42)	0.000
University	249	121 (48.6)	128 (51.4)	0.63 (0.37–1.06)	0.085	0.43 (0.24–0.79)	0.006
Household average monthly income (US $)							
1–50	495	171 (34.5)	324 (65.5)	1		1	
51–250	669	321 (48.0)	348 (52.0)	1.75 (1.37–2.22)	0.000	1.74 (1.35–2.24)	0.000
251–500	79	54 (68.4)	25 (31.6)	4.09 (2.46–6.81)	0.000	3.89 (2.29–6.64)	0.000
501–1,000	26	25 (96.2)	1 (3.8)	47.4 (6.36–352)	0.000	54.2 (7.12–413)	0.000
Occupation							
Unemployed	134	56 (41.8)	78 (58.2)	1		1	
Housewife	240	86 (35.8)	154 (64.2)	0.78 (0.50–1.20)	0.255	1.13 (0.69–1.84)	0.624
Student	231	113 (48.9)	118 (51.1)	1.33 (0.87–2.04)	0.189	1.79 (1.07–3.01)	0.026
Public servant	40	23 (57.5)	17 (42.5)	1.88 (0.92–3.85)	0.082	2.28 (1.06–4.93)	0.035
Private sector	403	181 (44.9)	222 (55.1)	1.13 (0.76–1.69)	0.528	1.37 (0.88–2.14)	0.154
Healthcare worker	221	112 (50.7)	109 (49.3)	1.43 (0.93–2.21)	0.105	1.58 (0.96–2.61)	0.072
Religion							
Christian	1,110	493 (44.4)	617 (55.6)	1		1	
Traditional	115	59 (51.3)	56 (48.7)	1.32 (0.89–1.94)	0.158	1.47 (0.97–2.24)	0.068
Other	44	19 (43.2)	25 (56.8)	0.95 (0.52–1.75)	0.872	1.04 (0.54 2.01)	0.906
Knowledge							
Poor	251	74 (29.5)	177 (70.5)	1		1	
Good	1,018	497 (48.8)	521 (51.2)	2.28 (1.69–3.07)	0.000	2.49 (1.82–3.43)	0.000

### Attitude toward COVID-19

Almost 40% of respondents had a positive attitude, while 36.6% had a negative attitude, and 23.5% had a neutral attitude toward the assessed COVID-19 preventive measures. More than half of respondents (54.5%) were willing to get a COVID-19 test if they experienced flu-like symptoms, while only 40.2% were willing to get the COVID-19 vaccine (Table 5). We found a trend toward increased odds of positive attitude regarding COVID-19 preventive measures with age. Respondents aged 29–39 years, 40–49 years, and more than 50 years were, respectively, 1.5 (AOR 1.50, 95% CI 1.04–2.19, *p* = 0.031), 2.1 (AOR 2.07, 95% CI 1.39–3.08, *p* = 0.000) and 2.4 times (AOR 2.38, 95% CI 1.62–3.50, *p* = 0.000) more likely to have a positive attitude than those 18–28 years of age (Table 6). Similarly, respondents with good knowledge were 55% more likely to have a good attitude (AOR 1.55, 95% CI 1.15–2.08, *p* = 0.003). However, respondents with primary or vocational education were less likely to have a positive attitude than respondents with no formal education. They had, respectively, 54 and 59% decreased odds of positive attitude (AOR 0.46, 95% CI 0.24–0.91, *p* = 0.025 and AOR 0.41, 95% CI 0.21–0.80, *p* = 0.009).

**Table 5:** Participants’ attitudes toward COVID-19 preventive measures.

Attitudes questions (*N* = 1,269)	*n*	%
Willingness to get tested in case of flu-like symptoms		
Not willing	88	6.9
Somewhat not willing	101	7.9
Undecided	203	16.0
Somewhat willing	186	14.7
Willing	691	54.5
Willingness to get the COVID-19 vaccine		
Not willing	202	15.9
Somewhat not willing	153	12.1
Undecided	250	19.7
Somewhat willing	154	12.1
Willing	510	40.2

**Table 6:** Factors associated with participants’ attitude toward COVID-19 preventive measures.

Variables (*N* = 1,269)	*N*	Attitude		OR	*p*	AOR	*p*
		Positive, *n* (%)	Negative, *n* (%)				
Sex							
Female	774	467 (60.3)	307 (39.7)	1		1	
Male	495	338 (68.3)	157 (31.7)	1.41 (1.12–1.80)	0.004	1.17 (0.89–1.53)	0.240
Health district							
Matadi	733	452 (61.7)	281 (38.3)	1		1	
Nzanza	536	353 (65.9)	183 (34.1)	1.20 (0.95–1.51)	0.126	1.08 (0.84–1.41)	0.508
Age group in years							
18–28	437	240 (54.9)	197 (45.1)	1		1	
29–39	274	170 (62.0)	104 (38.0)	1.34 (0.99–1.83)	0.062	1.50 (1.04–2.19)	0.031
40–49	248	171 (68.9)	77 (31.1)	1.82 (1.31–2.53)	0.000	2.07 (1.39–3.08)	0.000
> 50	310	224 (72.3)	86 (27.7)	2.14 (1.57–2.92)	0.000	2.38 (1.62–3.50)	0.000
Education							
No formal education	75	55 (73.3)	20 (26.7)	1		1	
Primary	111	65 (58.6)	46 (41.4)	0.51 (0.27–0.97)	0.040	0.46 (0.24–0.91)	0.025
Secondary	688	421 (61.2)	267 (38.8)	0.57 (0.34–0.97)	0.041	0.57 (0.32–1.01)	0.054
Vocational	146	83 (56.9)	63 (43.1)	0.48 (0.26–0.88)	0.018	0.41 (0.21–0.80)	0.009
University	249	181 (72.7)	68 (27.3)	0.97 (0.54–1.73)	0.913	0.89 (0.47–1.67)	0.725
Household average monthly income (US $)							
1–50	495	310 (62.6)	185 (37.4)	1		1	
51–250	669	420 (62.8)	249 (37.2)	1.00 (0.79–1.28)	0.957	0.89 (0.69–1.15)	0.386
251–500	79	49 (62.0)	30 (38.0)	0.97 (0.60–1.59)	0.918	0.82 (0.49–1.38)	0.450
501–1,000	26	26 (100.0)	0 (0.0)				
Occupation							
Unemployed	134	83 (61.9)	51 (38.1)	1		1	
Housewife	240	134 (55.8)	106 (44.2)	0.78 (0.50–1.20)	0.252	0.85 (0.53–1.36)	0.490
Student	231	139 (60.2)	92 (39.8)	0.93 (0.60–1.44)	0.739	1.37 (0.83–2.27)	0.215
Public servant	40	32 (80.0)	8 (20.0)	2.46 (1.05–5.75)	0.038	1.82 (0.75–4.44)	0.183
Private sector	403	259 (64.3)	144 (35.7)	1.11 (0.74–1.66)	0.627	1.15 (0.75–1.79)	0.515
Healthcare worker	221	158 (71.5)	63 (28.5)	1.54 (0.98–2.43)	0.062	1.28 (0.77–2.12)	0.344
Religion							
Christian	1,110	714 (64.3)	396 (35.7)	1		1	
Traditional	115	69 (60.0)	46 (40.0)	0.83 (0.56–1.23)	0.359	0.99 (0.66–1.49)	0.968
Other	44	22 (50.0)	22 (50.0)	0.55 (0.30–1.01)	0.056	0.56 (0.29–1.06)	0.073
Knowledge							
Poor	251	134 (53.4)	117 (46.6)	1		1	
Good	1,018	671 (65.9)	347 (34.1)	1.69 (1.27–2.23)	0.000	1.55 (1.15–2.08)	0.003

### Practice of government-related COVID-19 preventive measures

Most respondents (80.8%) had poor practice, whereas only 5.8% had good practice of COVID-19 preventive measures when the survey was conducted. Compared to early phases of the pandemic, facemask wearing was still the most practiced preventive measure at the time of data collection despite a slight decrease. The proportion of respondents practicing measures such as staying at home, avoiding crowded spaces, keeping a 1.5 m distance, washing clothes upon returning home, or reducing the use of public transportation decreased by nearly half at the time of data collection than during early phases of the pandemic (Table 7). The mean practice score during the survey period (3.22 ± 2.28) was lower as opposed to earlier phases of the pandemic (4.63 ± 2.51), with a statistically significant decrease in score of 1.40 (95% CI 1.25–1.55, *p* < 0.000).

**Table 7:** Participants’ practice of government-related COVID-19 preventive measures.

Practice questions (*N* = 1,269) Are you practicing any of the following government recommendations to stop the spread of COVID-19?	Practiced during the early phases of the pandemic		Practiced during the survey period	
	*n*	%	n	%
*Facemask wearing*				
No	57	4.5	165	13.0
Yes	1,212	95.5	1,104	87.0
*Gloves wearing*				
No	1,105	87.1	1,168	92.0
Yes	164	12.9	101	8.0
*Hand washing with soap*				
No	291	22.9	406	32.0
Yes	978	77.1	863	68.0
*Hand washing at least 20 s*				
No	419	33.0	534	42.1
Yes	850	67.0	735	57.9
*Staying at home*				
No	691	54.5	1,009	79.5
Yes	578	45.5	260	20.5
*Avoiding crowded spaces*				
No	595	46.9	923	72.7
Yes	674	53.1	346	27.3
*Keeping 1.5 m distance from each other*				
No	584	46.0	898	70.8
Yes	685	54.0	371	29.2
*Washing clothes upon returning home*				
No	979	77.2	1,127	88.8
Yes	290	22.8	142	11.2
*Reducing the use of public transportation*				
No	821	64.7	1,093	86.1
Yes	448	35.3	176	13.9

Regarding COVID-19 vaccination status, only 18/1,269 (1.4%) respondents had received at least one dose of vaccine, most of whom were healthcare workers 8/18 (44.4%).

The multivariable analysis showed that respondents from the Nzanza health district were 43% less likely to practice COVID-19 preventive measures than those from the Matadi health district (AOR 0.57, 95% CI 0.41–0.81, *p* = 0.002; Table 8). Similarly, students were 53% less likely to practice COVID-19 preventive measures than unemployed respondents (AOR 0.47, 95% CI 0.24–0.95, *p* = 0.034). On the contrary, respondents with primary education were twice as likely to practice COVID-19 preventive measures than those without formal education (AOR 2.77, 95% CI 1.21–6.38, *p* = 0.016). Respondents living in households with an average monthly income of US $ 51–250 were nearly twice as likely to practice COVID-19 preventive measures than respondents from households with an average monthly income of US $ 1–50 (AOR 1.79, 95% CI 1.28–2.52, *p* = 0.001).

**Table 8:** Factors associated with participants’ practice of COVID-19 preventive measures.

Variables (*N* = 1,269)	*N*	Practice		OR	*p*	AOR	*p*
		Good, *n* (%)	Poor, *n* (%)				
Sex							
Female	774	155 (20.0)	619 (80.0)	1		1	
Male	495	89 (18.0)	406 (82.0)	0.87 (0.65–1.17)	0.367	0.83 (0.59–1.17)	0.290
Health district							
Matadi	733	162 (22.1)	571 (77.9)	1		1	
Nzanza	536	82 (15.3)	454 (84.7)	0.64 (0.47–0.85)	0.003	0.57 (0.41–0.81)	0.002
Age group in years							
18–28	437	63 (14.4)	374 (85.6)	1		1	
29–39	274	42 (15.3)	232 (84.7)	1.07 (0.70–1.64)	0.739	0.87 (0.51–1.48)	0.612
40–49	248	59 (23.8)	189 (76.2)	1.85 (1.25–2.75)	0.002	1.15 (0.69–1.92)	0.588
> 50	310	80 (25.8)	230 (74.2)	2.06 (1.43–2.98)	0.000	1.40 (0.85–2.29)	0.192
Education							
No formal education	75	12 (16.0)	63 (84.0)	1		1	
Primary	111	41 (36.9)	70 (63.1)	3.07 (1.48–6.37)	0.002	2.77 (1.21–6.38)	0.016
Secondary	688	119 (17.3)	569 (82.7)	1.09 (0.57–2.09)	0.777	1.31 (0.62–2.79)	0.481
Vocational	146	20 (13.7)	126 (86.3)	0.83 (0.38–1.81)	0.646	0.80 (0.32–1.98)	0.634
University	249	52 (20.9)	197 (79.1)	1.38 (0.69–2.76)	0.353	1.46 (0.64–3.30)	0.368
Household average monthly income (US $)							
1–50	495	72 (14.5)	423 (85.5)	1		1	
51–250	669	161 (24.1)	508 (75.9)	1.86 (1.37–2.52)	0.000	1.79 (1.28–2.52)	0.001
251–500	79	8 (10.1)	71 (89.9)	0.66 (0.31–1.43)	0.295	0.44 (0.19–1.00)	0.051
501–1,000	26	3 (11.5)	23 (88.5)	0.77 (0.22–2.62)	0.671	0.57 (0.16–2.13)	0.409
Occupation							
Unemployed	134	32 (23.9)	102 (76.1)	1		1	
Housewife	240	52 (21.7)	188 (78.3)	0.88 (0.53–1.46)	0.623	1.02 (0.56–1.85)	0.942
Student	231	28 (12.1)	203 (87.9)	0.44 (0.25–0.77)	0.004	0.47 (0.24–0.95)	0.034
Public servant	40	7 (17.5)	33 (82.5)	0.67 (0.27–1.67)	0.398	0.46 (0.17–1.24)	0.125
Private sector	403	72 (17.9)	331 (82.1)	0.69 (0.43–1.11)	0.128	0.65 (0.38–1.13)	0.132
Healthcare worker	221	53 (24.0)	168 (76.0)	1.01 (0.61–1.66)	0.983	1.03 (0.56–1.88)	0.931
Religion							
Christian	1,110	222 (20.0)	888 (80.0)	1		1	
Traditional	115	18 (15.6)	97 (84.4)	0.74 (0.44–1.25)	0.265	0.67 (0.37–1.19)	0.177
Other	44	4 (9.1)	40 (90.9)	0.40 (0.14–1.12)	0.084	0.43 (0.14–1.33)	0.143
Knowledge							
Poor	251	6 (2.4)	245 (97.6)	1		1	
Good	1,018	238 (23.4)	780 (76.6)	12.4 (5.47–28.4)	0.000	12.5 (5.41–28.8)	0.000
Perception							
Poor	698	93 (13.3)	605 (86.7)	1		1	
Good	571	151 (26.4)	420 (73.6)	2.34 (1.76–3.12)	0.000	1.81 (1.31–2.52)	0.000
Attitude							
Negative	464	63 (13.6)	401 (86.4)	1		1	
Positive	805	181 (22.5)	624 (77.5)	1.85 (1.35–2.52)	0.000	1.48 (1.04–2.11)	0.028

Regarding the influence of knowledge, attitude, and perception on practice, respondents with good knowledge, positive attitude or good perception were, respectively, 12.5 (AOR 12.5, 95% CI 5.41–28.8, *p* = 0.000), 1.5 (AOR 1.48, 95% CI 1.04–2.11, *p* = 0.028), and 1.8 (AOR 1.81, 95% CI 1.31–2.52, *p* = 0.000) times more likely to practice COVID-19 preventive measures than respondents with poor knowledge, negative attitude or poor perception (Table 8).

## Discussion

We conducted a cross-sectional study to assess the knowledge and perception of government-related COVID-19 preventive measures, attitudes, and practices among adult residents of Matadi and to determine associated factors. Our results indicate that 75.6% of respondents had good knowledge of the assessed COVID-19 preventive measures. Studies conducted in Ethiopia and Uganda earlier during the pandemic reported similar results (14, 21, 22). However, this finding is slightly lower than one could expect since the study was conducted by the end of the third epidemic wave after several awareness campaigns on COVID-19 prevention were implemented by the local COVID-19 response team. A high proportion of respondents (76.8%) had heard about the COVID-19 vaccine nearly 5 months after the vaccination campaign started, highlighting the effectiveness of awareness campaigns on vaccines as a pillar for COVID-19 prevention. Conversely, only 36.3% of respondents knew the nearest COVID-19 testing center. This result underscores the poor health service utilization resulting from limited testing capacities, the stigma and misconception about COVID-19.

The likelihood of good knowledge of preventive measures increased with age, especially for respondents aged 40–49. This is consistent with studies conducted in Ethiopia, Spain, and Uganda, where older age was associated with good knowledge (14, 21–23). Respondents aged at least 40 are more likely to be exposed to information and have the adequate background to process it, partly because they might have experienced or heard of previous outbreaks. Similarly, the likelihood of good knowledge increased with education level, especially for respondents with vocational training. Several studies have reported a similar trend (13, 14, 21, 22). People with high education level know how critical information is and are more exposed to information through various channels (e.g., social media, websites, community events) either passively or actively.

Government-related preventive measures were perceived as efficient by 45.0% of respondents. Similar results have been reported in other countries, with slight variations depending on the level of trust in governments and public hospitals (24–26). However, in our study, the more respondents were educated, the less likely they perceived government measures as efficient. A study in 12 Latin American countries reported consistent results (27). The odds of perceiving preventive measures as efficient increased with the household average monthly income and being a student or public servant. Similarly, a multi-country study found that individuals from higher socioeconomic or educational status were less likely to have misperceptions about COVID-19 and government-related interventions (28). Our study population’s sociodemographic and cultural characteristics can explain the observed differences. Several approaches may improve the population’s perception, such as delivering clear, simple, positive, and consistent preventive messages using visual aids and several channels to reach a wider audience.

Regarding attitudes toward preventive measures, only 40% of respondents had a positive attitude. Similar results have been reported in DRC and Ethiopia (13, 15). Conversely, other studies in Ethiopia and Uganda reported nearly twice the proportion of respondents with a positive attitude (21, 22). Differences could be explained by respondents’ relatively higher education level and the study period. Studies reporting higher proportions of positive attitudes were conducted earlier during the pandemic when stringent public health measures were in place, and the fear of getting infected was high. Our study was conducted after three epidemic waves were recorded in DRC, with decreasing case fatality rates and thus decreasing perception of risk (29). There was a trend of reduced odds of positive attitude with education level especially for respondents with primary or vocational education. This is in line with a previous study conducted in DRC, which reported lower adherence to preventive measures among respondents with low education levels (13). These groups should be particularly targeted with tailored messages convened by peers. As reported by other studies, we found a trend toward increasing odds of positive attitude with age (15, 30). As for knowledge, the older the respondent gets, the more he is exposed to information, and the more likely his attitude will be positive.

Respondents willing to get COVID-19 vaccinated represented 54.5%. A previous study conducted a year earlier in seven provinces of the DRC reported COVID-19 vaccine acceptance rates of 55.9 and 52.0%, respectively, for all study sites and the Kongo Central province (16). Vaccine acceptance rates seemed not to have changed as the fear arising from the lack of local data on safety, rumors and conspiracy theories continued to prevail. In contrast, a longitudinal study across four waves in South Africa revealed that only 6.6% of respondents remained firmly vaccine-hesitant between survey waves (31). The large-scale vaccine rollout in health care workers contributed to sustaining respondents’ willingness to get vaccinated across survey waves despite blood clotting issues reported after the vaccination rollout (31). Our survey was conducted about 2 months after the launch of the vaccination campaign in Matadi, which started with healthcare workers as a strategy to increase population trust while protecting frontline workers. In fact, only 1.4% (18/1,269) of respondents had received at least one dose of the COVID-19 vaccine, with healthcare workers representing 44% (8/18). However, by the end of 2022, less than 4% of the DRC population had been fully vaccinated (32). Vaccine hesitancy, external aid-dependent supply, and logistic challenges have contributed to sustaining poor vaccination coverage (32). Additionally, population perception of risk has decreased over epidemic waves due to decreasing case fatality from 5.1% during the first wave to 0.9% during the fourth wave (29). Context-specific risk communication and community engagement strategies are needed for effective vaccine rollout or any other epidemic control intervention. Research and development capacities should be strengthened in African countries to cope with dependency on external supply and provide critical products that will be financially accessible and socially accepted.

Eight respondents out of 10 (80.8%) poorly practiced preventive measures. Higher proportions of respondents with better practice patterns have been reported in several studies (14, 15, 21, 22, 30). Studies reporting better practice patterns were conducted earlier during the pandemic when stringent preventive measures were in place and enforced by the law. In fact, compared to the pandemic’s early phases, our study’s mean practice score significantly decreased by 1.40. Furthermore, the proportion of respondents still practicing the assessed preventive measures decreased by half compared to the early phases of the pandemic, except for facemask wearing that nearly nine respondents out of 10 still practiced after the third epidemic wave. This finding could also reflect the progressively decreasing availability and accessibility to critical prevention resources such as masks, hand sanitizers, vaccines or testing kits. Surprisingly, respondents with a primary education level were twice as likely to practice preventive measures. Although some studies have reported an association between a higher level of education and higher odds of preventive measures practice (33, 34), a study conducted in Spain found results consistent with ours (23). Respondents with higher education may have been overloaded with information about COVID-19, causing pandemic fatigue and decreasing interest in practicing preventive measures. Therefore, preventive messages should be adapted over time to account for the long-lasting epidemic’s social and mental impact. We found that students were 53% less likely to practice preventive measures than unemployed respondents. Younger individuals and students have been disproportionately affected by COVID-19 through its impact on their daily lives and social interactions (35, 36). The frustration and psychological distress arising from prolonged restrictive measures may have contributed to decreasing their adherence to preventive measures in the long run. Youth-friendly support programs could address this issue and increase adherence to preventive measures during future epidemics. Respondents with a good knowledge of preventive measures were 12 times more likely to practice them. Similarly, respondents with a good attitude toward preventive measures were 48% more likely to practice them. This finding is in keeping with results from other studies reporting good knowledge and good attitude as determinants of good practice (15, 22, 23).

Our results revealed that although most respondents had good knowledge of COVID-19 preventive measures, they did not perceive them as efficient in controlling the disease spread, resulting in negative attitudes and poor practices. Noncompliers were mainly young, educated and with a lower socio-economic status. Noncompliance with COVID-19 preventive measures is a multifaceted concept with social, economic, psychological and political determinants. As previously reported, participants in this study failed to comply with recommendations, most likely due to pandemic fatigue and the exacerbation of pre-existing socioeconomic inequities and disparities (37). The latter may not have facilitated the emergence of social identity and accountability necessary for a collective response (38). Moreover, most respondents had lowered perceptions of risk because of the decreasing case fatality over epidemic waves and distrust of the government as a result of growing beliefs in conspiracy theories, especially on COVID-19 vaccines (38, 39). Finally, the decreasing availability and accessibility to critical tools and interventions for compliance (face masks, hand sanitizers, etc.) might have strengthened skepticism among residents. These determinants should be considered to enhance compliance and develop tailored and efficient countermeasures for future emergencies.

Our study has the merit of a robust sampling frame that yielded a large and representative sample of adult residents from Matadi. As such, it provides evidence supporting the need for adjusting current and future epidemic control strategies. However, respondents’ knowledge, attitude, practice, and perception patterns may have been rapidly evolving during the pandemic course, making the study’s cross-sectional design not optimal to capture trends over time and further elucidate the cause-effect relationship between the assessed variables. There may have been respondent bias through either exaggeration of answers or information retainment, especially for government-related questions. Finally, although logistic regression could accurately predict factors associated with good knowledge, positive attitude, good practice and good perception, other more robust machine learning techniques could have generated predictors of compliance to government preventive measures with more accuracy and precision as reported by some studies (40–43).

## Conclusion

Our study has shown that by the end of the third COVID-19 epidemic wave, Matadi residents had a high level of knowledge, a good perception of government-related COVID-19 preventive measures, a moderately positive attitude and an extremely low level of good practice. Age, education level, health district, profession, average household monthly income, good knowledge, positive attitude, and good perception were identified as main determinants. Despite the moderate adherence to vaccination as a pilar for COVID-19 control, vaccine uptake was extremely low. These results underscore the need to develop and implement context-based and risk group-specific communication and community engagement strategies to respond more efficiently to future outbreaks. These strategies should include refining messages considering emotions, values, and beliefs, fostering discussions and community activities such as showcasing positive role models. As the COVID-19 response is moving from emergency to control, and variants of concern with the potential of resisting previously acquired protection are still emerging, tailored and targeted interventions are needed more than ever to sustain the current disease control. Lessons learned from the current epidemic will improve preparedness and response to future outbreaks.

## Data availability statement

The data analyzed in this study is subject to the following licenses/restrictions: Data are available from the corresponding author upon reasonable request and approval by the Kinshasa School of Public Health Ethics Committee. Requests to access these datasets should be directed to SM-M, shemakiala@yahoo.fr.

## Ethics statement

The studies involving humans were approved by Kinshasa School of Public Health Ethics Committee. The studies were conducted in accordance with the local legislation and institutional requirements. The participants provided their written informed consent to participate in this study.

## Author contributions

YM-B: Conceptualization, Data curation, Formal analysis, Funding acquisition, Methodology, Project administration, Resources, Software, Supervision, Validation, Visualization, Writing – original draft, Writing – review & editing. PO-T: Investigation, Resources, Writing – original draft, Writing – review & editing. JN-M: Conceptualization, Data curation, Funding acquisition, Investigation, Methodology, Project administration, Resources, Supervision, Writing – original draft, Writing – review & editing. PM-I: Conceptualization, Funding acquisition, Methodology, Writing – original draft, Writing – review & editing. PT-M: Investigation, Resources, Writing – original draft, Writing – review & editing. AU-P: Investigation, Methodology, Resources, Supervision, Writing – original draft, Writing – review & editing. JK: Conceptualization, Resources, Supervision, Writing – original draft, Writing – review & editing. FA-P: Investigation, Methodology, Resources, Supervision, Writing – original draft, Writing – review & editing. GM: Conceptualization, Methodology, Resources, Writing – original draft, Writing – review & editing. RM-N: Conceptualization, Funding acquisition, Investigation, Methodology, Project administration, Resources, Writing – original draft, Writing – review & editing. RA: Conceptualization, Funding acquisition, Methodology, Project administration, Resources, Writing – original draft, Writing – review & editing. SM: Funding acquisition, Methodology, Project administration, Resources, Writing – original draft, Writing – review & editing, Conceptualization. SA-M: Writing – original draft, Writing – review & editing, Project administration, Funding acquisition, Conceptualization. MI: Conceptualization, Funding acquisition, Methodology, Project administration, Resources, Supervision, Writing – original draft, Writing – review & editing. SM-M: Conceptualization, Data curation, Funding acquisition, Investigation, Methodology, Project administration, Resources, Supervision, Validation, Writing – original draft, Writing – review & editing. J-JM-T: Conceptualization, Funding acquisition, Methodology, Project administration, Resources, Supervision, Writing – original draft, Writing – review & editing.

## References

[1] Khan S Siddique R Bai Q Shabana LY Xue M Nabi G. (COVID-19): causative agent, mental health concerns, and potential management options. J Infect Public Health. (2019) 13:1840–4. doi: 10.1016/j.jiph.2020.07.010 PMC7381894 32741731

[2] WHO Coronavirus (COVID-19) Dashboard. *WHO coronavirus (COVID-19) dashboard with vaccination data*. (2023). Available at: https://covid19.who.int/ (Accessed March 9, 2023).

[3] Bwire G Ario AR Eyu P Ocom F Wamala JF Kusi KA. The COVID-19 pandemic in the African continent. BMC Med. (2022) 20:1–23. doi: 10.1186/s12916-022-02367-4 35501853 PMC9059455

[4] Hafner M Yerushalmi E Fays C Dufresne E Van Stolk C. COVID-19 and the cost of vaccine nationalism. RAND Corp. (2020) 2020:769. doi: 10.7249/RRA769-1 PMC9519117 36238007

[5] Riaz MMA Ahmad U Mohan A dos Santos Costa AC Khan H Babar MS. Global impact of vaccine nationalism during COVID-19 pandemic. Trop Med Health. (2021) 49:101. doi: 10.1186/s41182-021-00394-0 34963494 PMC8714455

[6] Udoakang A Oboh M Henry-Ajala A Anyigba C Omoleke S Amambua-Ngwa A. Low COVID-19 impact in Africa: the multifactorial Nexus. Open Res Africa. (2021) 4:47. doi: 10.12688/AASOPENRES.13261.1

[7] Xylogiannopoulos KF Karampelas P Alhajj R. COVID-19 pandemic spread against countries’ non-pharmaceutical interventions responses: a data-mining driven comparative study. BMC Public Health. 21:1607. doi: 10.1186/s12889-021-11251-4 PMC8409702 34470630

[8] Teachout M Zipfel C. *The economic impact of COVID-19 lockdowns in sub-Saharan Africa: Policy brief*. (2020). Available at: https://www.wider.unu.edu/publication/estimates-impact-covid-19-global-poverty (Accessed March 8, 2023).

[9] Anyanwu JC Salami AO. The impact of COVID-19 on African economies: an introduction. Afr Dev Rev. (2021) 33:S1. doi: 10.1111/1467-8268.12531 34149237 PMC8207010

[10] Munyeku-Bazitama Y Folefack GT Yambayamba MK Tshiminyi PM Kazenza BM Otshudiema JO. High SARS-CoV-2 seroprevalence after second wave (October 2020–April 2021), Democratic Republic of the Congo. Emerg Infect Dis. (2023) 29:89–97. doi: 10.3201/eid2901.221009 36573545 PMC9796206

[11] Munyeku-Bazitama Y Okitale-Talunda P Mpingabo-Ilunga P Yambayamba MK Tshiminyi PM Umba-Phuati A. High severe acute respiratory syndrome coronavirus 2 antibody prevalence after the third epidemic wave (may–October 2021) in Matadi, Democratic Republic of the Congo. Open Forum Infect Dis. (2023) 10:23. doi: 10.1093/ofid/ofad023 PMC9887263 36726537

[12] Akilimali PZ Mashinda DK Lulebo AM Mafuta EM Onyamboko MA Tran NT. The emergence of COVID-19 in the Democratic Republic of Congo: community knowledge, attitudes, and practices in Kinshasa. PLoS One. (2022) 17:e0265538. doi: 10.1371/journal.pone.0265538 35727797 PMC9212135

[13] Ditekemena JD Nkamba DM Muhindo HM Siewe JNF Luhata C Van Den Bergh R. Factors associated with adherence to COVID-19 prevention measures in the Democratic Republic of the Congo (DRC): results of an online survey. BMJ Open. (2021) 11:e043356. doi: 10.1136/bmjopen-2020-043356 PMC7813390 33462101

[14] Feleke BT Wale MZ Yirsaw MT. Knowledge, attitude and preventive practice towards COVID-19 and associated factors among outpatient service visitors at Debre Markos compressive specialized hospital, north-West Ethiopia, 2020. PLoS One. (2021) 16:e0251708. doi: 10.1371/journal.pone.0251708 34264940 PMC8282282

[15] Bukata IT Dadi LS Ayana AM Mengistu D Yewal D Gizaw TS. Knowledge, attitudes, and practice toward prevention of COVID-19 among Jimma town residents: a community-based cross-sectional study. Front Public Heal. (2022) 10:942. doi: 10.3389/fpubh.2022.822116 PMC9091554 35570967

[16] Ditekemena JD Nkamba DM Mutwadi A Mavoko HM Siewe Fodjo JN Luhata C. COVID-19 vaccine acceptance in the Democratic Republic of Congo: a cross-sectional survey. Vaccine. (2021) 9:153. doi: 10.3390/vaccines9020153 PMC7917589 33672938

[17] Nkuba AN Makiala SM Guichet E Tshiminyi PM Bazitama YM Yambayamba MK. High prevalence of anti–severe acute respiratory syndrome coronavirus 2 (anti–SARS-CoV-2) antibodies after the first wave of coronavirus disease 2019 (COVID-19) in Kinshasa, Democratic Republic of the Congo: results of a cross-sectional household-based Su. Clin Infect Dis. (2022) 74:882–90. doi: 10.1093/cid/ciab515 34089598 PMC8244674

[18] United Nations Office for the Coordination of Humanitarian Affaires. *RDC - Statistiques des populations par zones de santé - humanitarian data exchange*. (2022). Available at: https://data.humdata.org/dataset/rdc-statistiques-des-populations (Accessed July 4, 2022).

[19] World Population Review. *Matadi population 2022 (demographics, maps, graphs)*. (2022). Available at: https://worldpopulationreview.com/world-cities/matadi-population (Accessed July 8, 2022).

[20] Bergeri I Lewis HC Subissi L Nardone A Valenciano M Cheng B. Early epidemiological investigations: World Health Organization UNITY protocols provide a standardized and timely international investigation framework during the COVID-19 pandemic. Influenza Other Respir Viruses. (2022) 16:7–13. doi: 10.1111/irv.12915 34611986 PMC8652791

[21] Ssebuufu R Sikakulya FK Mambo SB Wasingya L Nganza SK Ibrahim B. Knowledge, attitude, and self-reported practice toward measures for prevention of the spread of COVID-19 among Ugandans: a Nationwide online cross-sectional survey. Front Public Health. (2020) 8:731. doi: 10.3389/fpubh.2020.618731 PMC7793670 33425842

[22] Kedir S Yesse M Muze M Argaw B Dengo M Nesre T. Determinants of knowledge, attitude and practice towards preventive measures of COVID-19 among adult residencies in Silte zone, southern Ethiopia. Pan Afr Med J. (2022) 41:826. doi: 10.11604/pamj.2022.41.211.33826 PMC9167470 35721629

[23] González-Herrera A Rodríguez-Blázquez C Romay-Barja M Falcon-Romero M Ayala A Forjaz MJ. Age differences in knowledge, attitudes and preventive practices during the COVID-19 pandemic in Spain. Sci Rep. (2022) 12:1–8. doi: 10.1038/s41598-022-25353-5 36460702 PMC9718463

[24] Chu Q Gu T Li A Chen J Wang H Liu N. Perceived effectiveness of public health measures and positive attitudes during a pandemic: a large cross-sectional study in Shanghai. China BMJ Open. (2021) 11:e047231. doi: 10.1136/bmjopen-2020-047231 PMC8166628 34049920

[25] Georgieva I Lantta T Lickiewicz J Pekara J Wikman S Loseviča M. Perceived effectiveness, restrictiveness, and compliance with containment measures against the Covid-19 pandemic: an international comparative study in 11 countries. Int J Environ Res Public Health. (2021) 18:3806. doi: 10.3390/ijerph18073806 33917334 PMC8038651

[26] Mækelæ MJ Reggev N Dutra N Tamayo RM Silva-Sobrinho RA Klevjer K. Perceived efficacy of COVID-19 restrictions, reactions and their impact on mental health during the early phase of the outbreak in six countries. R Soc Open Sci. (2020) 7:200644. doi: 10.1098/rsos.200644 32968525 PMC7481706

[27] Mejia CR Liendo-Venegas D García-Gamboa F Mejía-Rodríguez MA Valladares-Garrido MJ. Factors associated with the perception of inadequate sanitary control in 12 Latin American countries during the COVID-19 pandemic. Front Public Health. (2022) 10:2272. doi: 10.3389/fpubh.2022.934087 PMC9358134 35958835

[28] Bhuiya T Klares R Conte MA Cervia JS. Predictors of misperceptions, risk perceptions, and personal risk perceptions about COVID-19 by country, education and income. J Investig Med. (2021) 69:1473–8. doi: 10.1136/jim-2021-001835 34380630

[29] Otshudiema JO Folefack GLT Nsio JM Mbala-Kingebeni P Kakema CH Kosianza JB. Epidemiological comparison of four COVID-19 waves in the Democratic Republic of the Congo, march 2020–January 2022. J Epidemiol Glob Health. (2022) 12:316–27. doi: 10.1007/s44197-022-00052-6 35921045 PMC9346056

[30] Desalegn Z Deyessa N Teka B Shiferaw W Hailemariam D Addissie A. COVID-19 and the public response: knowledge, attitude and practice of the public in mitigating the pandemic in Addis Ababa. Ethiopia PLoS One. (2021) 16:e0244780. doi: 10.1371/journal.pone.0244780 33411766 PMC7790293

[31] Burger R Köhler T Golos AM Buttenheim AM English R Tameris M. Longitudinal changes in COVID-19 vaccination intent among south African adults: evidence from the NIDS-CRAM panel survey, February to May 2021. BMC Public Health. (2022) 22:422. doi: 10.1186/s12889-022-12826-5 35236319 PMC8889513

[32] Zola Matuvanga T Doshi RH Muya A Cikomola A Milabyo A Nasaka P. Challenges to COVID-19 vaccine introduction in the Democratic Republic of the Congo–a commentary. Hum Vaccin Immunother. (2022) 18:272. doi: 10.1080/21645515.2022.2127272 PMC9746480 36165731

[33] Bekele F Sheleme T Fekadu G Bekele K. Patterns and associated factors of COVID-19 knowledge, attitude, and practice among general population and health care workers: a systematic review. SAGE Open Med. (2020) 8:97072. doi: 10.1177/2050312120970721 PMC7675903 33240497

[34] Wake AD. Knowledge, attitude, practice, and associated factors regarding the novel coronavirus disease 2019 (COVID-19) pandemic. Infect Drug Resist. (2020) 13:3817–32. doi: 10.2147/IDR.S275689 33149627 PMC7603646

[35] Devita M Di Rosa E Iannizzi P Bianconi S Contin SA Tiriolo S. Cognitive and psychological sequelae of COVID-19: age differences in facing the pandemic. Front. Psychiatry. (2021) 12:1461. doi: 10.3389/fpsyt.2021.711461 PMC8481580 34603102

[36] Romay-Barja M Pascual-Carrasco M De Tena-Dávila MJ Falcón M Rodriguez-Blazquez C Forjaz MJ. How patients with COVID-19 managed the disease at home during the first wave in Spain: a cross-sectional study. BMJ Open. (2021) 11:e048702. doi: 10.1136/bmjopen-2021-048702 PMC8137165 34016666

[37] Cowling BJ Ng DMW Ip DKM Liao Q Lam WWT Wu JT. Community psychological and behavioral responses through the first wave of the 2009 influenza a(H1N1) pandemic in Hong Kong. J Infect Dis. (2010) 202:867–76. doi: 10.1086/655811 20677945

[38] Fancourt D Steptoe A Wright L. The cummings effect: politics, trust, and behaviours during the COVID-19 pandemic. Lancet. (2020) 396:464–5. doi: 10.1016/S0140-6736(20)31690-1 32771083 PMC7613216

[39] Uluşahin Y Mavor K Reicher S. A political psychology of the link between populist beliefs and compliance with COVID-19 containment measures. Front. Pol Sci. (2024) 6:798. doi: 10.3389/fpos.2024.1279798

[40] Pavlović T Azevedo F De K Riaño-Moreno JC Maglić M Gkinopoulos T. Predicting attitudinal and behavioral responses to COVID-19 pandemic using machine learning. PNAS Nexus. (2022) 1:1–15. doi: 10.1093/pnasnexus/pgac093 PMC9381137 35990802

[41] Pradhan AK Mishra D Das K Obaidat MS Kumar M. A COVID-19 X-ray image classification model based on an enhanced convolutional neural network and hill climbing algorithms. Multimed Tools Appl. (2023) 82:14219–37. doi: 10.1007/s11042-022-13826-8 36185320 PMC9513301

[42] Raheja S Kasturia S Cheng X Kumar M. Machine learning-based diffusion model for prediction of coronavirus-19 outbreak. Neural Comput & Applic. (2023) 35:13755–74. doi: 10.1007/s00521-021-06376-x PMC8358916 34400853

[43] Aggarwal A Chakradar M Bhatia MS Kumar M Stephan T Gupta SK. COVID-19 risk prediction for diabetic patients using fuzzy inference system and machine learning approaches. J Healthc Eng. (2022) 2022:1–10. doi: 10.1155/2022/4096950 PMC8974235 35368915

